# Supplementary material for: FERN – a Java framework for stochastic simulation and evaluation of reaction networks
Source: BMC Bioinformatics. 2008 Aug 29;9:356. doi: 10.1186/1471-2105-9-356 (PMC2553347; doi:10.1186/1471-2105-9-356)
Supplement: Additional file 1 — FERN distribution, Version 1.3. This archive contains the FERN source code and binaries as well as documentation and example models in FernML and SBML. [file 1471-2105-9-356-S1.zip › fern/doc/javadoc/index-files/index-17.html]

S-Index


---


|  |  |  |
| --- | --- | --- |
| **Overview** \| Package \| Class \| Use \| **Tree** \| **Deprecated** \| **Index** \| **Help** | |  |
| **PREV LETTER**   **NEXT LETTER** | **FRAMES**    **NO FRAMES**     **All Classes** |  |


A B C D E F G H I J L M N O P R S T U V 

---


## **S**

**save()** - Method in class fern.network.AmountManager: Makes a copy of the amount array. **saveData(File)** - Method in class fern.tools.gnuplot.GnuPlot: Saves the gnuplot data to one ore more files. **saveHistogram(Map<Integer, Integer>, File)** - Static method in class fern.tools.NumberTools: Writes a histogram to a file. **saveImage(File)** - Method in class fern.tools.gnuplot.GnuPlot: Saves the plotted data to a png file. **saveToFile(File)** - Method in class fern.network.fernml.FernMLNetwork: Saves the actual `FernMLNetwork` to a file. **saveToFile(File)** - Method in class fern.network.sbml.SBMLNetwork: Saves the current `SBMLNetwork` to a file. **saveToFile(File, long, long)** - Method in class fern.network.sbml.SBMLNetwork: Saves the current `SBMLNetwork` to a sbml file with given level and version. **SBaseAdded(PluginSBase)** - Method in class fern.cellDesigner.FernCellDesignerPlugin: **SBaseChanged(PluginSBase)** - Method in class fern.cellDesigner.FernCellDesignerPlugin: **SBaseDeleted(PluginSBase)** - Method in class fern.cellDesigner.FernCellDesignerPlugin: **SBMLEventHandlerObserver** - Class in fern.network.sbml: Observer which handles an event of a sbml model. **SBMLEventHandlerObserver(Simulator, SBMLNetwork, Event)** - Constructor for class fern.network.sbml.SBMLEventHandlerObserver: Creates the observer. **SBMLMathTreeTest** - Class in fern.example: **SBMLMathTreeTest()** - Constructor for class fern.example.SBMLMathTreeTest: **SBMLNetwork** - Class in fern.network.sbml: For specifications of the sbml format refer to http:\\www.sbml.org. **SBMLNetwork(File)** - Constructor for class fern.network.sbml.SBMLNetwork: Creates a network from a sbmlfile. **SBMLNetwork(File, boolean)** - Constructor for class fern.network.sbml.SBMLNetwork: Creates a network from a sbml file. **SBMLNetwork(Network)** - Constructor for class fern.network.sbml.SBMLNetwork: Create a `SBMLNetwork` from an existing `Network`. **SBMLPropensityCalculator** - Class in fern.network.sbml: Propensity calculator which is used for `SBMLNetwork`s. **SBMLPropensityCalculator(SBMLNetwork)** - Constructor for class fern.network.sbml.SBMLPropensityCalculator: Creates the `MathTree`s and parses the parameters. **search(IntSearchStructure, int[], int[], NetworkSearchAction)** - Method in class fern.analysis.AnalysisBase: Performs a search starting at the given sources (which means the contents of `speciesSource` and `reactionSource` are the initial content of the search structure `IntSearchStructure`. **sep** - Static variable in class fern.cellDesigner.ui.ExtendedPane: **sep** - Static variable in class fern.cytoscape.ui.ExtendedPane: **setAmount(int, long)** - Method in class fern.network.AmountManager: Sets the current amount of a species. **setAmount(int, long)** - Method in class fern.simulation.algorithm.GibsonBruckSimulator: **setAmount(int, long)** - Method in class fern.simulation.algorithm.GillespieEnhanced: **setAmount(int, long)** - Method in class fern.simulation.algorithm.HybridMaximalTimeStep: **setAmount(int, long)** - Method in class fern.simulation.Simulator: Sets the amount of the given species. **setAmountBottomColor(Color)** - Method in class fern.cytoscape.ColorCalculator: **setAmountTopColor(Color)** - Method in class fern.cytoscape.ColorCalculator: **setAttributes(NetworkChecker)** - Method in class fern.cytoscape.ui.ExtendedPane: **setCatalyzedKineticConstant(double)** - Method in class fern.network.creation.AutocatalyticNetwork: Sets the kinetic constant for catalyzed reactions. **setColor(Node, Color)** - Method in class fern.cytoscape.CytoscapeColorChangeObserver.ColorChangingNodeAppeareanceCalculator: **setConstant(int, double)** - Method in class fern.network.ArrayKineticConstantPropensityCalculator: **setCountGenerations(boolean)** - Method in class fern.tools.Stochastics: Sets whether or not to count the number of random number generations **setDefaultStyle(String)** - Method in class fern.tools.gnuplot.GnuPlot: The defaultStyle is used, when no style is defined for an column in an axes object. **setEfficientlyAdaptSum(boolean)** - Method in class fern.simulation.algorithm.GillespieEnhanced: **setEnabled(boolean)** - Method in class fern.cellDesigner.ui.ExtendedPane: **setEnabled(boolean)** - Method in class fern.cellDesigner.ui.OverviewPane: **setEnabled(boolean)** - Method in class fern.cytoscape.ui.ExtendedPane: **setEnabled(boolean)** - Method in class fern.cytoscape.ui.OverviewPane: **setEnabledTauLeaping(boolean)** - Method in class fern.cellDesigner.ui.ExtendedPane: **setEnabledTauLeaping(boolean)** - Method in class fern.cytoscape.ui.ExtendedPane: **setEpsilon(double)** - Method in class fern.simulation.algorithm.AbstractBaseTauLeaping: The epsilon is an error control parameter and bounds the expected change of the propensity functions by epsilon \* a\_sum. **setErrorMessage(Network, String)** - Method in class fern.cellDesigner.ui.OverviewPane: **setErrorMessage(Network, String)** - Method in class fern.cytoscape.ui.OverviewPane: **setInitialAmount(int, long)** - Method in class fern.cellDesigner.CellDesignerNetworkWrapper: **setInitialAmount(int, long)** - Method in class fern.cytoscape.CytoscapeNetworkWrapper: **setInitialAmount(int, long)** - Method in class fern.network.creation.AutocatalyticNetwork: **setInitialAmount(int, long)** - Method in class fern.network.fernml.FernMLNetwork: **setInitialAmount(int, long)** - Method in class fern.network.modification.ExtractSubNetwork: **setInitialAmount(int, long)** - Method in class fern.network.modification.ModifierNetwork: **setInitialAmount(int, long)** - Method in interface fern.network.Network: Sets the initial amount of the specified molecule species. **setInitialAmount(int, long)** - Method in class fern.network.sbml.SBMLNetwork: **setInterpolateTheta(boolean)** - Method in class fern.simulation.Simulator: Sets whether the amount values are returned interpolated for theta events. **setLabel(int, String)** - Method in class fern.tools.gnuplot.Axes: Sets the label for a column. **setLabelFormat(String)** - Method in class fern.simulation.observer.Observer: Sets the label format for `Observer.getLabel(String)`. **setLangevinThreshold(double)** - Method in class fern.simulation.algorithm.AbstractBaseTauLeaping: The Langevin threshold determines, when the normal distribution is used as an approximation for the poisson distribution. **setMonomerAmount(long)** - Method in class fern.network.creation.AutocatalyticNetwork: Sets the initial amount of the monomers for a simulation algorithm. **setNCritical(int)** - Method in class fern.simulation.algorithm.AbstractBaseTauLeaping: The nCritical determines, when a reaction is called critical. **setNetworkAnnotation(String, String)** - Method in class fern.cytoscape.CytoscapeAnnotationManager: **setNetworkAnnotation(String, String)** - Method in interface fern.network.AnnotationManager: Sets the network annotation of the specified field. **setNetworkAnnotation(String, String)** - Method in class fern.network.AnnotationManagerImpl: **setNetworkAnnotation(String, String)** - Method in class fern.network.fernml.FernMLAnnotationManager: **setNetworkChecker(NetworkChecker)** - Method in class fern.cytoscape.FernVisualStyle: **setNumBins(int)** - Method in class fern.benchmark.Benchmark: Sets the number of bins that are used for creating gnuplot histograms. **setNumSimpleCalls(int)** - Method in class fern.simulation.algorithm.AbstractBaseTauLeaping: The numSimpleCalls determines, how often the SSA is called, when tau leaping is abandoned. **setOtherAmount(long)** - Method in class fern.network.creation.AutocatalyticNetwork: Sets the initial amount of the not-monomers for a simulation algorithm. **setPlotQuality(boolean)** - Method in class fern.simulation.observer.IntervalObserver: Sets whether or not to plot quality data. **setPrintWriter(PrintWriter)** - Method in class fern.simulation.observer.Observer: Registers a `PrintWriter` **setReactionAnnotation(int, String, String)** - Method in class fern.cytoscape.CytoscapeAnnotationManager: **setReactionAnnotation(int, String, String)** - Method in interface fern.network.AnnotationManager: Sets the reaction annotation of the specified field. **setReactionAnnotation(int, String, String)** - Method in class fern.network.AnnotationManagerImpl: **setReactionAnnotation(int, String, String)** - Method in class fern.network.fernml.FernMLAnnotationManager: **setReactionColor(Color)** - Method in class fern.cytoscape.ColorCalculator: **setReactionFire(Node)** - Method in class fern.cytoscape.FernVisualStyle: **setReactionUnFire(Node)** - Method in class fern.cytoscape.FernVisualStyle: **setRecordStart(double)** - Method in class fern.simulation.observer.ReactionIntervalObserver: Sets the moment in time where the recording starts **setScale(ColorCalculator.Scale)** - Method in class fern.cytoscape.ColorCalculator: **setScaleMax(double)** - Method in class fern.cytoscape.ColorCalculator: **setSeed(long)** - Method in class fern.tools.Stochastics: Sets a new seed for the random number generator. **setSeed(Date)** - Method in class fern.tools.Stochastics: Sets a new seed for the random number generator. **setShowSteps(int)** - Method in class fern.benchmark.SimulatorPerformance: Sets the number of iterations between two `present`-calls. **setSimulator(Simulator)** - Method in class fern.simulation.observer.Observer: Sets the simulator. **setSimulatorAsync(Simulator)** - Method in class fern.network.sbml.SBMLEventHandlerObserver: **setSpecies(Network)** - Method in class fern.cellDesigner.ui.ExtendedPane: **setSpecies(Network)** - Method in class fern.cytoscape.ui.ExtendedPane: **setSpeciesAnnotation(int, String, String)** - Method in class fern.cytoscape.CytoscapeAnnotationManager: **setSpeciesAnnotation(int, String, String)** - Method in interface fern.network.AnnotationManager: Sets the species annotation of the specified field. **setSpeciesAnnotation(int, String, String)** - Method in class fern.network.AnnotationManagerImpl: **setSpeciesAnnotation(int, String, String)** - Method in class fern.network.fernml.FernMLAnnotationManager: **setSpeciesInitialAmount(Node, long)** - Method in class fern.cytoscape.NetworkChecker.NodeParameter: **setStyle(int, String)** - Method in class fern.tools.gnuplot.Axes: Sets the style for a column. **setTheta(double)** - Method in class fern.simulation.observer.Observer: Sets the moment in time, where the simulator has to invoke `Observer.theta(double)`. **setThetaMethod(boolean)** - Method in class fern.simulation.observer.IntervalObserver: Using theta method means that the time intervals are registered at the simulator and the simulator invokes at this moment the method theta. **setTime(double)** - Method in class fern.simulation.controller.DefaultController: Sets the time where the simulation has to stop. **setUncatalyzedKineticConstant(double)** - Method in class fern.network.creation.AutocatalyticNetwork: Sets the kinetic constant for not catalyzed reactions. **setUseSimpleFactor(double)** - Method in class fern.simulation.algorithm.AbstractBaseTauLeaping: The useSimpleFactor determines, when the tau leaping is abandoned and the SSA method is used (if tau**setValue(Node, double, double)** - Method in class fern.cytoscape.FernVisualStyle: **setVerbose(boolean)** - Method in class fern.simulation.algorithm.AbstractBaseTauLeaping: Sets if each step is to print to stdout. **setVolume(double)** - Method in class fern.simulation.algorithm.GibsonBruckSimulator: **setVolume(double)** - Method in class fern.simulation.algorithm.GillespieEnhanced: **setVolume(double)** - Method in class fern.simulation.algorithm.HybridMaximalTimeStep: **setVolume(double)** - Method in class fern.simulation.Simulator: Sets the volume of the reaction network. **ShortestPath** - Class in fern.analysis: Computes shortest paths in the network by a bfs. **ShortestPath(Network)** - Constructor for class fern.analysis.ShortestPath: Creates the class with the specified network. **ShortestPath.Path** - Class in fern.analysis: Encapsulates a path from one species to another. **ShortestPath.Path(int[])** - Constructor for class fern.analysis.ShortestPath.Path: Creates a path from an array. **shuffle(int[])** - Static method in class fern.tools.NumberTools: Shuffles the content of the given array. **sigma** - Variable in class fern.simulation.algorithm.AbstractTauLeapingPropensityBoundSimulator: **SimulationController** - Interface in fern.simulation.controller: `Simulator`s are controlled by implementing classes. **Simulator** - Class in fern.simulation: Base class for stochastic simulators. **Simulator(Network)** - Constructor for class fern.simulation.Simulator: Creates a new simulator for the given network. **Simulator.FireType** - Enum in fern.simulation: Defines different types of a firing for reactions. **SimulatorCorrectness** - Class in fern.benchmark: Benchmark the correctness of the built-in simulators for a given net. **SimulatorCorrectness(Network, double, String...)** - Constructor for class fern.benchmark.SimulatorCorrectness: Creates the benchmark instance with given network, moment in time and species. **SimulatorFireTypes** - Class in fern.benchmark: Benchmark the `Simulator.FireType`s for a given net. **SimulatorFireTypes(Network, double)** - Constructor for class fern.benchmark.SimulatorFireTypes: Create the benchmark and defines the time each simulator has to run in one iteration. **simulatorNames** - Variable in class fern.benchmark.SimulatorPerformance: Contains the names of the simulators for using in the `present`-method of extending classes. **SimulatorPerformance** - Class in fern.benchmark: Measures the performance of the different simulation algorithms for a given network. **SimulatorPerformance(Network)** - Constructor for class fern.benchmark.SimulatorPerformance: Registers the six built-in simulators for the performance benchmarks. **SimulatorRandomNumbers** - Class in fern.benchmark: Check the number of random number creations of different distributions for a given net. **SimulatorRandomNumbers(Network, double)** - Constructor for class fern.benchmark.SimulatorRandomNumbers: Create the benchmark and defines the time each simulator has to run in one iteration. **simulators** - Variable in class fern.benchmark.SimulatorPerformance: Contains the `Simulator`s - use this field to attach `Observer`s. **SimulatorTime** - Class in fern.benchmark: **SimulatorTime(Network, double)** - Constructor for class fern.benchmark.SimulatorTime: Create the benchmark and defines the time each simulator has to run in one iteration. **size()** - Method in class fern.simulation.algorithm.IndexedPriorityQueue: Gets the number of nodes in the binary heap. **speciesDiscovered(int)** - Method in interface fern.analysis.NetworkSearchAction: Gets called when a species is inserted into the search structure. **speciesFinished(int)** - Method in interface fern.analysis.NetworkSearchAction: Gets called when a species gets out of the search structure. **speciesIdToIndex** - Variable in class fern.network.AbstractNetworkImpl: Stores a mapping from species names to their indices. **start()** - Method in class fern.benchmark.Benchmark: sets a start time for the benchmark system **start(double)** - Method in class fern.simulation.Simulator: Starts the simulation up to a given time. **start(SimulationController)** - Method in class fern.simulation.Simulator: Start the simulation with the given `SimulationController`. **Start** - Class in fern: **Start()** - Constructor for class fern.Start: **started()** - Method in class fern.cytoscape.CytoscapeColorChangeObserver: **started()** - Method in class fern.example.CellGrowthObserver: **started()** - Method in class fern.network.sbml.SBMLEventHandlerObserver: **started()** - Method in class fern.simulation.observer.AmountAtMomentObserver: Register theta at the simulator. **started()** - Method in class fern.simulation.observer.FireTypeObserver: Do nothing. **started()** - Method in class fern.simulation.observer.InstantOutputObserver: Prints event. **started()** - Method in class fern.simulation.observer.IntervalObserver: Clears the recorded values and registers (if needed) `theta` **started()** - Method in class fern.simulation.observer.LeapObserver: Clears the recorded data. **started()** - Method in class fern.simulation.observer.Observer: Gets called when the simulation has started after the initialization and before the termination condition is checked the first time. **started()** - Method in class fern.simulation.observer.RandomNumberGeneratorCallObserver: Resets the count variables of the random number generator calls. **started()** - Method in class fern.simulation.observer.ReactionIntervalObserver: **stddev(double[])** - Static method in class fern.tools.NumberTools: Gets the standard deviation of the array **step()** - Method in class fern.cytoscape.CytoscapeColorChangeObserver: **step()** - Method in class fern.example.CellGrowthObserver: **step()** - Method in class fern.network.sbml.SBMLEventHandlerObserver: **step()** - Method in class fern.simulation.observer.AmountAtMomentObserver: Do nothing. **step()** - Method in class fern.simulation.observer.FireTypeObserver: Do nothing. **step()** - Method in class fern.simulation.observer.InstantOutputObserver: Prints event. **step()** - Method in class fern.simulation.observer.IntervalObserver: If the `step` method is used, the data is recorded here. **step()** - Method in class fern.simulation.observer.LeapObserver: Record the time and amounts of the species. **step()** - Method in class fern.simulation.observer.Observer: Gets called after each termination check and before `Simulator.performStep(fern.simulation.controller.SimulationController)` is called. **step()** - Method in class fern.simulation.observer.RandomNumberGeneratorCallObserver: Do nothing. **stochastics** - Variable in class fern.simulation.Simulator: Contains a shortcut to the `Stochastics` framework. **Stochastics** - Class in fern.tools: Manages a random number generators for various distributions. **sum(int[])** - Static method in class fern.tools.NumberTools: Gets the sum of the values in the array.

---


|  |  |  |
| --- | --- | --- |
| **Overview** \| Package \| Class \| Use \| **Tree** \| **Deprecated** \| **Index** \| **Help** | |  |
| **PREV LETTER**   **NEXT LETTER** | **FRAMES**    **NO FRAMES**     **All Classes** |  |


A B C D E F G H I J L M N O P R S T U V 

---
